# Pharmacy Data for Tuberculosis Surveillance and Assessment of Patient Management

**DOI:** 10.3201/eid1008.031075

**Published:** 2004-08

**Authors:** Deborah S. Yokoe, Steven W. Coon, Rachel Dokholyan, Michael C. Iannuzzi, Timothy F. Jones, Sarah Meredith, Marisa Moore, Lynelle Phillips, Wayne Ray, Stephanie Schech, Deborah Shatin, Richard Platt

**Affiliations:** * Brigham and Women's Hospital and Harvard Medical School, Boston, Massachusetts, USA;; † Henry Ford Health System, Detroit, Michigan, USA;; ‡ Harvard Pilgrim Health Care, Boston, Massachusetts, USA;; § Tennessee Department of Health, Nashville, Tennessee, USA;; ¶ Center for Education and Research in Therapeutics and Vanderbilt University, Nashville, Tennessee, USA;; # Centers for Disease Control and Prevention, Atlanta, Georgia, USA;; ** Missouri Department of Health and Senior Services, Jefferson City, Missouri, USA;; †† Center for Health Care Policy and Evaluation, Minneapolis, Minnesota, USA;; ‡‡ HMO Research Network Center for Education and Research on Therapeutics, Boston, Massachusetts, USA

**Keywords:** tuberculosis, Mycobacterium tuberculosis, population surveillance, prescriptions, drug, patient compliance, health maintenance organizations, managed care programs

## Abstract

Underreporting tuberculosis (TB) cases can compromise surveillance. We evaluated the contribution of pharmacy data in three different managed-care settings and geographic areas. Persons with more than two anti-TB medications were identified by using pharmacy databases. Active TB was confirmed by using state TB registries, medical record review, or questionnaires from prescribing physicians. We identified 207 active TB cases, including 13 (6%) missed by traditional surveillance. Pharmacy screening identified 80% of persons with TB who had received their medications through health plan–reimbursed sources, but missed those treated solely in public health clinics. The positive predictive value of receiving more than two anti-TB medications was 33%. Pharmacy data also provided useful information about physicians' management of TB and patients' adherence to prescribed therapy. Pharmacy data can help public health officials to find TB cases and assess their management in populations that receive care in the private sector.

## Abstract 2

Pharmacy data help locate tuberculosis cases and assess their management.

Controlling and preventing tuberculosis (TB) continue to be major public health challenges in the United States (1). Information obtained through TB surveillance ensures that TB-control activities are appropriate and can be used to evaluate the effectiveness of public health programs (2). Because TB surveillance relies heavily on laboratories and providers to report cases to local health departments, surveillance data can be compromised by underreporting, particularly by private-sector clinicians who treat TB infrequently. Pharmacy data, often available in automated form, may supplement traditional TB reporting, especially because anti-TB medications are rarely used to treat other conditions.

A Massachusetts study found that persons with TB who were identified through pharmacy dispensing records and who had not been previously reported to the state health department represented 16% of all new cases (3). In that study, receipt of two or more anti-TB drugs identified most cases of active TB. These results suggested that pharmacy dispensing information could supplement traditional TB surveillance. In addition, pharmacy dispensing information for persons with active TB provided useful information about appropriateness of prescribed treatment regimens and adherence to therapy (4).

We therefore evaluated the contribution of pharmacy data to overall TB surveillance and to assessing the quality of TB management. We performed this study through health plans to facilitate access to pharmacy dispensing data and medical records.

## Methods

### Study Population

Members of three different health plans in Michigan (1993–1999), Missouri (1996–1998), and Tennessee (1998) were included in the study population. Study periods were based on availability of pharmacy data.

All health plans met our basic criteria of providing most of the medical care to defined populations, providing prescription drug benefits, having automated pharmacy claims files, and having accessible full-text medical records. The health plans differed in some ways. Most importantly, plan C (see below) routinely delegated care of recognized TB patients to local health departments or had members obtain their anti-TB drugs from public health programs separate from the plan's regular pharmacy programs and data systems. Additionally, the structure of the three health plans and their populations differed; they consisted of the following: a mixed staff and group model that included a large urban population (plan A); an independent practice association (IPA) health plan affiliated with a managed-care organization, principally serving an employed population (plan B); and a mixed IPA and staff model, principally serving Medicaid enrollees (plan C). Staff-model health plans employ providers who practice in common facilities. IPA-model health plans contract with providers who practice in their own offices (5). Prior institutional review board approval was obtained from participating health plans.

### Pharmacy Screening

Health plan pharmacy dispensing data were screened to identify all members who received two or more anti-TB medications during the study period. For plans A, B, and C, we screened, respectively, approximately 1.3 million, 1.0 million, and 1.6 million health plan person-years. The anti-TB medications included in the screening were isoniazid, rifampin, pyrazinamide, ethambutol, streptomycin, ethionamide, kanamycin, cycloserine, capreomycin, para-aminosalicylic acid (PAS), and drugs containing any combination of these medications. Although at least 90% of health plan members had some form of pharmacy benefit, the plans varied considerably in directing their members to public health facilities to obtain anti-TB medications.

### Identifying TB Cases

Reporting confirmed or clinically suspected TB to local or state health departments by providers, laboratories, boards of health, or administrators of hospitals is mandatory in Michigan, Missouri, and Tennessee, which maintain registries of all verified cases. State health department staff in all three states determined whether health plan members identified as having received two or more anti-TB medications had been reported previously to the health departments by matching to the state TB registries by using previously described methods (6).

For all plan members who received two or more anti-TB medications and who were not previously reported to the state health departments, information was obtained through review of medical records. A case of TB was defined according to the Centers for Disease Control and Prevention (CDC) surveillance definition (7). In a culture-positive case, *Mycobacterium tuberculosis* was isolated from a clinical specimen. In a smear-positive case, acid-fast bacilli (AFB) were demonstrated in a specimen in the absence of a culture. A clinical case-patient met all of the following criteria: a positive tuberculin skin test, signs and symptoms compatible with TB, and treatment with two or more anti-TB drugs. Case-patients without a positive culture for *M. tuberculosis* that were not known to the health departments were verified by review with clinicians experienced in diagnosing and treating TB.

To estimate the number of TB cases not detected by using pharmacy data, each health plan's membership during the study period was matched to the state health department's TB registry entries during the same period by using minimal disclosure methods (6). Potential matches were confirmed with full identifiers. To determine the source of care for patients not identified through pharmacy screening, health department records of all such patients in plans A and B and a random sample in plan C were reviewed.

### Assessing TB Management

Automated pharmacy dispensing records were used to characterize TB therapy for persons with active TB who met pharmacy screening criteria in plans A and B. Plan C did not participate in the assessment of TB management because members were routinely referred to public health clinics for treatment, and information about medications was unavailable from the health plan pharmacy database. In addition, pharmacy dispensing records from two additional health plans affiliated with plan B were screened, and TB cases verified through medical record review were included in the analysis.

All filled prescriptions were identified for isoniazid, rifampin, pyrazinamide, ethambutol, streptomycin, ethionamide, kanamycin, cycloserine, capreomycin, PAS, and drugs containing a combination of these medications. Initial regimens, i.e., those dispensed at the start of therapy before susceptibility results were known, and final treatment regimens were graded for consistency with American Thoracic Society (ATS) and CDC guidelines in effect at the time of diagnosis (8). The appropriateness of doses based on patient weight was not evaluated.

Two measures were calculated for therapeutic adequacy. The standard regimen dispensed is a percentage calculated by comparing the cumulative dose of each drug dispensed with the total recommended. Each drug received equal weight to a maximum of 100% per drug, as noted in the following formula for a three-drug regimen: percent standard regimen = ([D_1_/SR_1_] + [D_2_/SR_2_] + [D_3_/SR_3_]) x (100/3), where D_X_ is the cumulative dose for drug X and SR_X_ is the recommended total dose. Patients with a score >80% were considered to have received an appropriate amount of anti-TB medication. The days without medication for isoniazid or another drug required for the duration of treatment are calculated by dividing the total number of days without medication (based on medication refill intervals and quantities dispensed) by the number of days between the first and last dispensing (4,9).

## Analysis

The sensitivity of pharmacy data was defined as the number of verified TB cases detected by pharmacy screening divided by the total number of verified TB cases identified through the TB registry, pharmacy data, or both methods. The positive predictive value (PPV) of pharmacy screening was defined as the number of verified TB cases detected by pharmacy data divided by the total number of persons meeting pharmacy screening criteria; persons with undetermined case status were excluded. Exact binomial confidence intervals were calculated for sensitivity and PPV (8).

## Results

### Dispensing Anti-TB Drugs

A total of 244 patients received two or more anti-TB drugs (Table 1). Of these, 13 (5%) met the TB case definition and had not been previously reported to their respective state health departments. Another 61 (25%) were active TB case-patients. Sixty-three percent did not meet the TB case definition, and the status of the remaining 7% could not be determined because the medical records were either unavailable or insufficient.

**Table 1:** Identification of tuberculosis (TB) cases by using pharmacy screening

Cases	Plan A (%)	Plan B (%)	Plan C (%)	Total (%)
Total no. dispensed 2 or more anti-TB drugs	73	28	143	244
Matched to TB registry (previously reported TB cases)	12 (17)	6 (21)	43 (30)	61 (25)
Previously unreported TB cases (verified by record review)	3 (4)	1 (4)	9 (6)^a^	13 (5)
Not a TB case (verified by record review)	55 (75)	7 (25)	91 (64)	153 (63)
Case status not determined	3 (4)	14 (50)	0	17 (7)

^a^Includes two cases not found in the state health department's TB registry but reported to other state health departments.

Of 153 patients who received at least two anti-TB medications but did not meet the CDC TB case definition, 62 (41%) were treated for suspected active TB. Of these, 15 (24%) received a full course of therapy for suspected active TB. Twenty-one (14%) received more than one drug during treatment for latent TB infection, 63 (41%) were treated for non-TB mycobacterial infections, and 7 (4%) were treated for noninfectious conditions or for unknown reasons (Table 2).

**Table 2:** Reasons for meeting pharmacy screening criteria among persons without active tuberculosis (TB)

Reasons why non-TB cases met screening criteria	Plan A (%)	Plan B (%)	Plan C (%)	Total (%)
Suspected active TB, full course of therapy	7 (13)	0	8 (9)	15 (10)
Suspected active TB, empiric therapy discontinued	12 (22)	0	35 (38)	47 (31)
Treatment of latent TB infection	8 (14)	3 (43)	10 (11)	21 (14)
Other mycobacterial infections	26 (47)	4 (57)	33 (36)	63 (41)
Other or unknown	2 (4)	0	5 (5)	7 (4)
Total	55	7	91	153

The overall rate of initiating two or more anti-TB drugs was 6 per 100,000 person-years, ranging from 3 to 11 per 100,000 person-years in the three health plans. Confirmed case rates ranged from 0.9 to 4.3 per 100,000 person-years screened. The 1998 TB incidence for the three states ranged from 3.4 to 8.1 cases per 100,000 persons (9). For persons meeting pharmacy screening criteria, the proportion confirmed as new case-patients did not vary significantly among the three plans (Table 1).

### Newly Identified Cases of TB

A total of 207 health plan members meeting TB case definitions (53 in plan A, 22 in plan B, and 132 in plan C) were identified through pharmacy data or health department records (Table 3). Among these, 13 case-patients (6%) were unknown to the respective state health departments. Two persons with TB unknown to one health department had been reported to Mississippi State Department of Health. None of the 13 were culture-positive for *M. tuberculosis*; one lacked a microbiology culture but met the smear-positive case definition, and the remaining 12 met the CDC TB clinical case definition. All except one involved active pulmonary disease.

**Table 3:** Detecting tuberculosis (TB) cases by using pharmacy screening and state health department TB registries

Case identification	Plan A (%)	Plan B (%)	Plan C (%)	Total (%)
Pharmacy screening only	3 (6)	1 (5)	9 (7)	13 (6)
State health department only (all cases)	38 (72)	15 (68)	80 (61)	133 (64)
State health department only (health plan–treated patients)	16 (52)	2 (22)	0^a^	18 (19)
Both methods	12 (22)	6 (27)	43 (32)	61 (30)
Total (all cases)	53	22	132	207
Total (health plan–treated patients^b^)	31	9	52	92

^a^Extrapolated from review of a random sample of 28 of the 80 TB cases identified by the state health department and not by pharmacy screening. ^b^Excludes TB patients receiving anti-TB medication from public health clinics; these medications are not included in the health plan pharmacy databases.

One hundred thirty-three TB cases were known to the state health departments but were not identified through pharmacy databases. We reviewed the records of 81 of these patients, of whom 61 (75%) received their anti-TB medications from public health clinics; this proportion ranged from 58% (22/38) in plan A to 93% in plan C (26/28). An additional 3 (4%) were treated at Veterans Administration (VA) facilities or were diagnosed with TB during hospitalization and died before discharge. Health plan medical records did not include information about TB diagnosis and treatment for 17 (21%) patients. Reasons for this may include TB treatment exclusively by other providers and incomplete documentation in accessible medical records.

**Figure 1:**
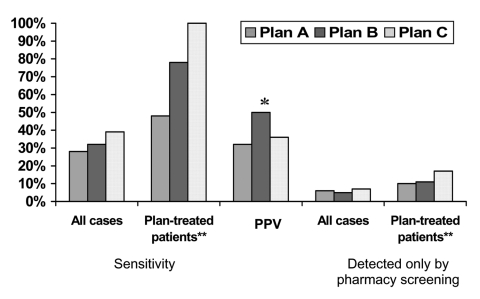
Sensitivity and positive predictive value (PPV) of pharmacy screening and percentage of tuberculosis (TB) cases detected only by pharmacy screening. *Of 28 members who met pharmacy screening criteria, TB case status was verified for 14. PPV calculation based on total of 14 with verified status. **Health plan–treated patients excludes patients receiving anti-TB medication from public health clinics.

The overall sensitivity of the pharmacy screening method to identify persons with active TB was 36% (28% in plan A, 32% in plan B, and 39% in plan C). However, the overall sensitivity was 80% after the extrapolated number of persons who received their TB medication from public health clinics rather than the health plans was excluded (Figure 1). The positive predictive value of the pharmacy screening method to identify persons with active TB was 33% (21% in plan A, 50% in plan B, and 36% in plan C) (Figure 1).

### Assessing Management of TB

Of the 29 plan A (n = 15) and plan B (n = 14) members with active TB identified through pharmacy screening, health plan and health department records indicated that 17 (59%) did not receive treatment in public health clinics and were likely to have received their anti-TB medications through health plan–reimbursed pharmacies. Twenty-eight (97%) patients received initial regimens through pharmacies reimbursed by the health plan. In all instances, the initial regimen dispensed was appropriate. For all 17 patients not treated in public health clinics, the final regimen described in the medical record was adequate with regard to the agents used, doses prescribed, and intended duration of treatment.

**Figure 2:**
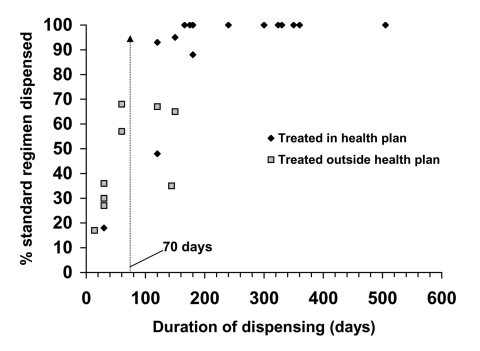
Pharmacy-dispensing profiles of tuberculosis (TB) case-patients treated in the health plans and at least partially outside the health plan. Percentage of standard regimen dispensed is plotted against duration of dispensing anti-TB medications for the two groups. A cutoff value of >70 days of medication dispensed from health plan–reimbursed pharmacies identifies all but one of the health plan–treated TB case-patients.

Fifteen of the 17 health plan–treated patients received anti-TB medications for at least 70 days (compared to 3 of 13 who were treated outside the health plans [relative risk = 3.8, p < 0.01]), with a median dispensing duration of 180 days (interquartile range 150–324 days). The median standard-regimen-dispensed score was 100% (interquartile range 93%–100%) (Figure 2). Based on health plan pharmacy data, one patient received an inadequate treatment regimen, with a standard-regimen-dispensed percentage of only 48%. Another health plan–treated patient received a standard-regimen-dispensed score of 100% but had a days-without-medication score of 51%, because of a gap in anti-TB therapy of 143 days. One additional patient with culture-positive *M. tuberculosis* infection received a standard-regime-dispensed score of 68% and a 60-day duration of dispensing. In all of these cases, the treating physician did not describe noncompliance or document a non–health plan source of anti-TB medications.

## Discussion

TB surveillance has traditionally depended on reporting by laboratories, public health clinics, hospitals, and private practitioners. Several retrospective studies (3,10–13) indicate that TB cases may be underreported, particularly those without positive cultures. In this study, we found that 6% of all TB cases in the three participating health plans had not been reported to state health departments. Most cases missed by traditional surveillance were culture- and smear-negative; however, nearly all patients with missed cases had clinical evidence of pulmonary disease and were therefore of public health interest.

The recent shift of populations at risk for TB, including Medicaid recipients, into managed care raises concerns about reporting. As the proportion of patients with TB who are cared for outside traditional public health–funded clinics grows, the benefit of adjunct surveillance methods based on pharmacy data is likely to increase, since these data are available for a large segment of the U.S. population.

Although we used health plan data for this study, health departments could more efficiently obtain this information directly from pharmacy benefits management companies (PBMs) that act as intermediaries between managed-care organizations and pharmacies, because they administer and manage the prescription drug benefit programs for these organizations. Working directly with PBMs has two advantages. First, PBM information is accessible in real time. Second, since the three largest PBMs in the United States manage the pharmacy claims of approximately 200 million persons, information available from a small number of PBMs could provide a rich resource for public health screening (14–17).

The percentage of cases in these three health plans that were missed by traditional surveillance (6%) was lower than the 16% missed in the Massachusetts study (3). This difference may reflect the fact that public health clinics cared for more patients in these health plans than in Massachusetts, where 60% of patients were treated solely by health plan providers, compared to about 40% for these health plans.

Patients who received their anti-TB medications from public health clinics were not identified through health plans' pharmacy data. However, because these patients are already known to the public health system, supplemental surveillance methods are unnecessary. Pharmacy screening identified 80% of the patients with TB who were not treated outside the health plan. This estimate is conservative; we probably underestimated the number of persons receiving anti-TB medications from public health clinics or other healthcare systems because we based this assessment on the private providers' records. Intermittent enrollment may also have compromised the sensitivity of pharmacy-based screening. Larger databases that include pharmacy information from multiple health plans within a geographic area, such as those maintained by PBMs, are likely to improve case-finding.

The most common reasons for dispensing two or more anti-TB medications to persons who did not meet the case definition were 1) more than one drug used to treat latent TB infection; 2) suspected active TB; 3) treatment of other mycobacterial infections; and 4) treatment for suspected active TB and receiving full courses of therapy, despite not meeting the CDC surveillance definition for TB, based on information available from their medical records. Persons in the last category may warrant additional evaluation by health departments because the case definition may not detect all patients who meet clinical standards for treatment.

The PPV of pharmacy screening criteria may be lower in clinical settings where treatment of non-TB mycobacterial infections is common. One strategy to increase the efficiency of pharmacy-based screening would be to use microbiologic culture information to quickly identify and exclude from further follow-up any persons with results indicating mycobacterial species other than *M. tuberculosis*. Complete laboratory reporting for *M. tuberculosis* is an important prerequisite for efficiently implementing this surveillance strategy.

In routine practice, pharmacy data might be used for active TB case-finding, with direct reporting from organizations dispensing drug information, such as health plans or PBMs, to local or state health departments. These data are typically available from health plans within 1 or 2 months and from PBMs within a day. Such reporting would require verifying case status by health department personnel.

Obtaining and reviewing medical records for this study were labor-intensive, but collecting this information from providers in real time should be more efficient. The cost of reporting would be relatively small for health plans or pharmacy benefits managers, and the cost per person identified would be small for large organizations. The additional costs for health departments to evaluate the status of persons not already identified will vary considerably across health departments. Despite the increased emphasis on privacy, current laws specifically allow reports of protected health information to support public health activities.

Although pharmacy data may be useful, they will not replace traditional surveillance of suspected and confirmed TB cases. Because rapidly following-up suspected TB cases is essential to prevent the spread of *M. tuberculosis*, educating providers to report suspected cases promptly to public health officials will continue to be important.

Automated pharmacy data also provided useful information about physicians' management of TB and about patients' adherence to prescribed therapy. Monitoring these aspects of TB care is particularly important when care is decentralized or when patients receive care from more than one provider. Pharmacy information demonstrated that, in nearly all cases, appropriate empiric regimens were prescribed. In most cases managed by health plan providers, full ATS/CDC-recommended regimens were dispensed. Consistent with the Massachusetts study results, using a cutoff value of at least 70 days of therapy identified most patients treated solely within the health plan. This practice is important in monitoring adherence to therapy, since automated pharmacy information is complete only for these patients. Pharmacy data also identified several persons with evidence of suboptimal adherence to therapy. Pharmacy information on anti-TB drugs could thus be used for monitoring the appropriateness of case management and to evaluate the program.

This study and our earlier work demonstrate that pharmacy data may be useful in settings where TB care is provided by the private healthcare system. Centralized repositories of pharmacy data, such as those maintained by PBMs, may facilitate even more efficient application of this surveillance strategy to find TB cases and assess TB management for large patient populations. Similar studies in other settings could expand our understanding of current surveillance limitations and provide better estimates of the true burden of TB in the United States. Similar strategies could also be considered to augment traditional surveillance for other diseases of public health importance.

This study was supported by Department of Health and Human Services, Centers for Disease Control and Prevention, Cooperative Agreement R18/CCU115960 and by the HMO Research Network Center for Education and Research in Therapeutics (CERT), Agency for Healthcare Research and Quality Cooperative Agreement HS10391.
